# Anatomical differences of the vertebrobasilar artery between normal subjects and patients with cerebral infarction

**DOI:** 10.1097/MD.0000000000039105

**Published:** 2024-08-09

**Authors:** Geo-seong Park, Jung-soo Park

**Affiliations:** a Department of Neurosurgery, Jeonbuk National University Hospital, Jeonju-si, Jeollabuk-do Province, Korea; b Research Institute of Clinical Medicine of Jeonbuk National University, Jeonju-si, Jeollabuk-do Province, Korea; c Biomedical Research Institute of Jeonbuk National University Hospital, Jeonju-si, Jeollabuk-do Province, Korea.

**Keywords:** basilar artery, cerebral infarction, vertebrobasilar artery

## Abstract

Previous studies have reported various anatomical differences in the cerebral artery between healthy subjects and patients with posterior circulation cerebral infarction. In particular, basilar artery angulation has been associated with posterior circulation cerebral infarction. We compared anatomical variations and the degree of anterior and lateral vertebrobasilar artery angulation and deviation to compare the incidence of cerebral infarction of healthy subjects and patients with posterior circulation cerebral infarction. We compared basilar artery anatomy using brain magnetic resonance angiography in 97 patients who underwent brain magnetic resonance angiography during health checkups at our hospital and in 92 patients diagnosed with posterior circulation cerebral infarction between 2012 and 2022. Anatomical variations, including fetal-type posterior cerebral artery, hypoplastic P1 segment, vertebrobasilar dolichoectasia, and dominant vertebral artery, as well as the degree of anterior and lateral deviation and angulation, were evaluated. Correlations between these variations and the occurrence of cerebral infarction were analyzed. The prevalence of hypoplastic P1 was significantly differences in patients with posterior circulation cerebral infarction (odds ratio: 5.655). Furthermore, patients with posterior circulation cerebral infarction exhibited more acute anterior and lateral angulation, as well as lateral deviation. Hypoplastic P1 and more acute anterior or lateral angulation of the vertebrobasilar artery are associated with increased frequency of cerebral infarction.

## 1. Introduction

The vertebrobasilar artery (VBA) supplies blood to the brainstem and the posterior cerebral artery (PCA), which is located anterior to the brainstem. The VBA naturally exhibits angulation, which may be affected by the shear force of blood flow originating from the vertebral arteries.^[1]^ The angulation point may therefore become deformed in advanced age due to the effects of continuous blood flow, and such deformation may cause intraplaque hemorrhage.^[2]^ Intraplaque hemorrhage associated with embolic infarction is observed exclusively in lesions in areas of low shear stress.^[3]^

Various studies have reported anatomical differences between healthy subjects and patients with cerebral infarctions, including vertebrobasilar dolichoectasia^[4]^ and differences in VBA angulation.^[2]^ We therefore investigated whether other anatomical differences are associated with the incidence of cerebral infarction.

## 2. Methods

This study was approved by the Institutional Review Board (IRB No. 2019-08-003-004).

**Figure 1.**
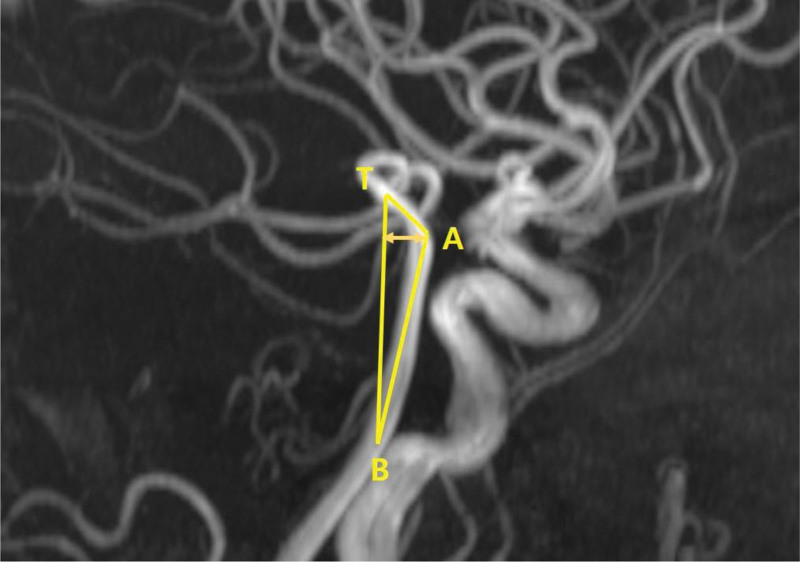
Calculation of anterior deviation (distance between TB and A) and angulation of the basilar artery (∠TAB).

**Figure 2.**
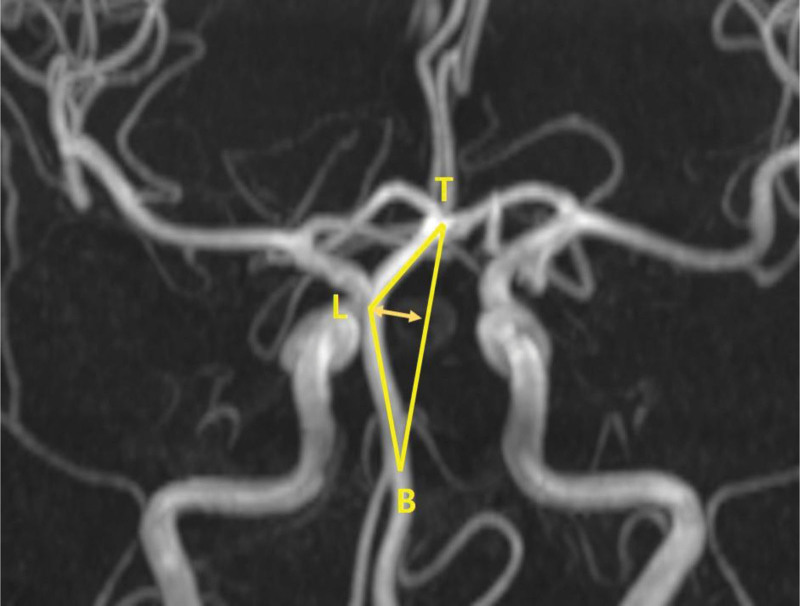
Calculation of lateral deviation (distance between TB and L) and angulation of the basilar artery (∠TLB).

We compared the anatomy of the VBA using brain magnetic resonance angiography (MRA). Between January 2012 and December 2022, 97 patients who underwent brain MRA during health checkups and 92 patients diagnosed with cerebral infarction in the emergency room were enrolled. Anatomical variations, including fetal-type PCA, hypoplastic P1 segment, vertebrobasilar dolichoectasia, and dominant vertebral artery (VA), were compared. The fetal-type PCA was defined as a complete fetal-type PCA that completely originates from the internal carotid artery with no connection with the basilar artery. Vertebrobasilar dolichoectasia was defined as a basilar artery diameter exceeding 4.5 mm,^[5]^, and dominant VA was defined as a side-to-side diameter difference ≥ 0.3 mm.^[6]^ The degree of anterior and lateral deviation was calculated as the distance between the most prominent point of the basilar artery (A and L) and the line between the top of the basilar artery (T) and the vertebrobasilar junction (B) (Figs. 1 and 2). The anterior and lateral angulation of the basilar artery was measured as the angle between the most prominent point of the basilar artery (A and L) and the line between the top of the basilar artery (T) and the vertebrobasilar junction (B) (Figs. 1 and 2).

The chi-square test was used to analyze anatomical variation. Logistic regression analyses were used to analyze differences in anterior and lateral deviation and angulation of the VBA between the control group and patients with cerebral infarction. Statistical analyses were performed using SPSS software, version 25 (SPSS Inc., Chicago, IL). Statistical significance was set as *P* < .05.

## 3. Results

In total, 97 healthy subjects and 92 patients with cerebral infarction were enrolled in this study. Patients in the control and cerebral infarction groups showed no significant differences in age or sex ratio. However, hyperlipidemia and the use of antiplatelet or anticoagulant agents due to comorbidities were more common in the cerebral infarction group (Table 1).

**Table 1:** Patient characteristics at baseline.

	Control group (n = 97)	Cerebral infarction group (n = 92)
Sex		
Male (n = 90)	45	45
Female (n = 99)	52	47
Age (yr)	71.44 ± 1.18	67.70 ± 1.54
Past history		
HTN (n = 94)	38	56
DM (n = 45)	23	22
Hyperlipidemia (n = 33)	6	27
Antiplatelet or anticoagulant (n = 73)	19	54

DM = diabetes mellitus, HTN = hypertension.

A significant difference was observed in the prevalence of a hypoplastic P1 segment between the control and cerebral infarction groups (Table 2), with a hypoplastic P1 segment independently associated with cerebral infarction (odds ratio [OR] = 5.655). However, the other vascular variations were not statistically significant (Table 3).

**Table 2:** Anatomical variations.

	Control group, n (%)		Cerebral infarction group, n (%)	
	Yes	No	Yes	No
Fetal-type PCA	83 (85.6)	14 (14.4)	77 (83.7)	15 (16.3)
Hypoplastic P1	78 (80.4)	19 (19.6)	88 (95.6)	4 (4.4)
Vertebrobasilar dolichoectasia	96 (99.0)	1 (1.0)	87 (94.6)	5 (5.4)
Dominant VA	71 (73.2)	26 (26.8)	60 (65.2)	32 (34.8)

PCA = posterior cerebral artery, VA = vertebral artery.

**Table 3:** Multivariate associations between the vascular variants and cerebral infarction.

	Cerebral infarction group (odds ratio)	*P*-value
Fetal-type PCA	—	0.840
Hypoplastic P1	5.655	0.001
Vertebrobasilar dolichoectasia	—	0.111
Dominant VA	—	0.271

PCA = posterior cerebral artery, VA = vertebral artery.

There were significant differences in anterior angulation, lateral deviation, and lateral angulation between the control and cerebral infarction groups (Table 4). Cerebral infarction was associated with anterior angulation (OR = 1.080), lateral deviation (OR = 1.717), and lateral angulation (OR = 1.112) (Table 5).

**Table 4:** Anterior and lateral deviation and angulation.

	Control group	Cerebral infarction group
Anterior deviation (mm)	2.94 ± 0.21	3.00 ± 0.23
Anterior angulation (°)	154.79 ± 1.70	96.87 ± 6.66
Lateral deviation (mm)	2.51 ± 0.30	3.09 ± 0.30
Lateral angulation (°)	157.74 ± 2.43	88.94 ± 6.76

**Table 5:** Multivariate associations between vertebrobasilar artery angulation and cerebral infarction.

	Cerebral infarction group (odds ratio)	*P*-value
Anterior deviation (mm)	1.207	0.463
Anterior angulation (°)	1.080	0.000
Lateral deviation (mm)	1.717	0.040
Lateral angulation (°)	1.112	0.002

## 4. Discussion

Anatomical variations in posterior circulation are frequent but are commonly asymptomatic. However, some anatomical variants, such as vertebrobasilar dolichoectasia, hypoplastic P1 segment, and fetal-type PCA, are positively correlated with ischemic posterior circulation stroke.^[5]^ Moreover, previous studies have identified changes in VBA morphology with aging with older patients exhibiting an acute angle, which is associated with deep pontine lacunar infarction.^[2]^ Tortuous vascularity, defined by angulation and deviation in this study, increases hemodynamic stress on the vertebrobasilar arterial wall, which can lead to vascular wall injury.^[1]^ In addition, the probability of an embolus occurring at an atherosclerotic lesion may increase due to turbulent flow, which is aggravated by a change in the blood flow vector due to more angulated vessels.^[7]^

In this study, a significant difference in the incidence of cerebral infarction was associated with the presence of the hypoplastic P1 segment. In addition, significant differences were observed in anterior and lateral angulations and lateral deviation, indicating that more tortuous vascularity is associated with an increased incidence of cerebral infarction. In particular, more acute anterior or lateral angulation of the VBA is strongly associated with increased cerebral infarction.

However, there seemed to be a positive correlation but no significant difference in fetal-type PCA, vertebrobasilar dolichoectasia, and dominant VA.

This study had a limited sample size and further multicenter studies with larger patient populations are required to verify these correlations. Furthermore, a larger study population and longer follow-up may reveal aging-related patterns of VBA changes and their correlation with cerebral infarction. Furthermore, we did not evaluate cerebral infarction caused by atherosclerotic lesions and by cardioembolic sources separately. Turbulent flow aggravated by VBA deviation and angulation, and resulting embolic infarction, may be attributed to anatomical differences; however, it is difficult to determine risk factors for cerebral infarction caused by cardioembolic sources. Additional studies categorizing the cause of infarction in patients diagnosed with posterior circulation cerebral infarction may identify various anatomical variations and the curvature of the VBA may serve as an independent risk factor.

## 5. Conclusion

Anatomical differences, specifically a hypoplastic P1 segment and more acute angulation of the anterior or lateral VBA, are associated with the incidence of cerebral infarction. According to this result, among patients who underwent brain MRA for the purpose of health checkup or visiting the emergency room, it is necessary to keep in mind that the possibility of cerebral infarction may be high in patients with hypoplastic P1 segment and acute angulation of the VBA.

## Author contributions

**Data curation:** Geo-seong Park.

**Formal analysis:** Geo-seong Park.

**Methodology:** Geo-seong Park.

**Software:** Geo-seong Park.

**Writing—original draft:** Geo-seong Park.

**Conceptualization:** Jung-soo Park.

**Supervision:** Jung-soo Park.

**Writing—review and editing:** Jung-soo Park.
